# Crystal structure of di­chlorido­bis­(dimethyl *N*-cyano­dithio­imino­carbonate)zinc

**DOI:** 10.1107/S2056989016002607

**Published:** 2016-02-24

**Authors:** Mouhamadou Birame Diop, Libasse Diop, Allen G. Oliver

**Affiliations:** a Laboratoire de Chimie Minérale et Analytique, Département de Chimie, Faculté des Sciences et Techniques, Université Cheikh Anta Diop, Dakar, Senegal; b Department of Chemistry and Biochemistry, University of Notre Dame, 246, Nieuwland, Science Hall, Notre Dame, IN 46557-5670, USA

**Keywords:** crystal structure, zinc chloride adduct, dimethyl *N*-cyano­dithio­imino­carbonate, tetra­hedral environment, layered structure

## Abstract

The Zn^II^ atom in the title complex, [ZnCl_2_(C_4_H_6_N_2_S_2_)_2_], is coordinated in a distorted tetra­hedral manner by two Cl atoms and two terminal N atoms of two dimethyl *N*-cyano­dithio­imino­carbonate ligands. In the crystal, the complex mol­ecules are connected through C—H⋯Cl hydrogen bonds and Cl⋯S contacts, leading to a two-dimensional structure extending parallel to the *ab* plane.

## Abstract 2

The title complex consists of a Zn^II^ atom coordinated by two Cl atoms and two dimethyl *N*-cyano­dithio­imino­carbonate ligands bonded through the terminal N atom in a distorted tetra­hedral manner. The complex mol­ecules inter­act through C—H⋯Cl and Cl⋯S inter­actions to give a layered structure in the crystal.

## Chemical context   

Two N and two S atoms in dimethyl *N*-cyano­dithio­imino­carbonate (DMCDIC), which are expected to act as hard and soft donors, respectively, according to Pearson’s concept, give an inter­esting coordination potential to this mol­ecule. However, only one structure of a metal complex with DMCDIC acting as a ligand has been reported (Kojić-Prodić *et al.*, 1992 ▸). Very recently, we reported the crystal structure of [CoCl_2_(DMCDIC)_2_] (Diop *et al.*, 2016 ▸). Because of the scarcity of data on the coordination ability of DMCDIC, we have focused on studying the inter­actions between some transition metal halides and this ligand, which has yielded the title complex.
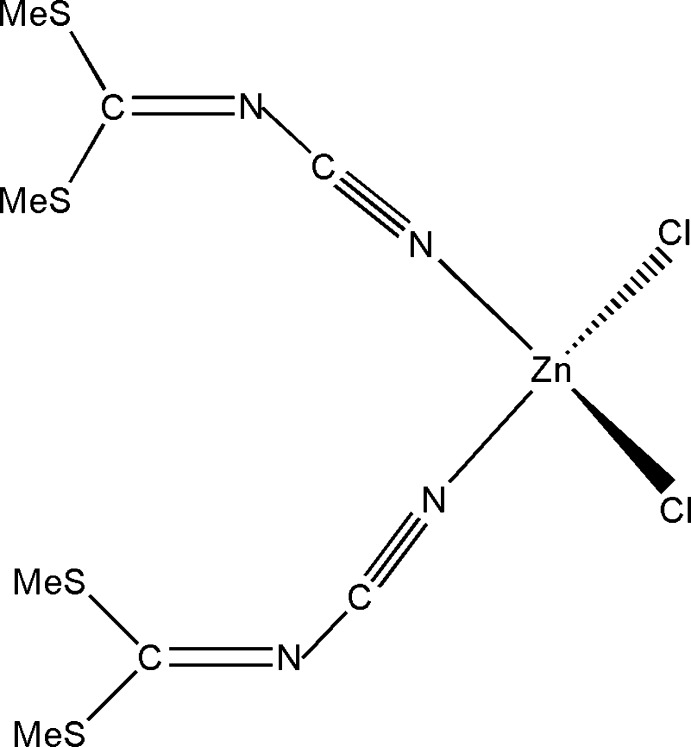


## Structural commentary   

**Figure 1:**
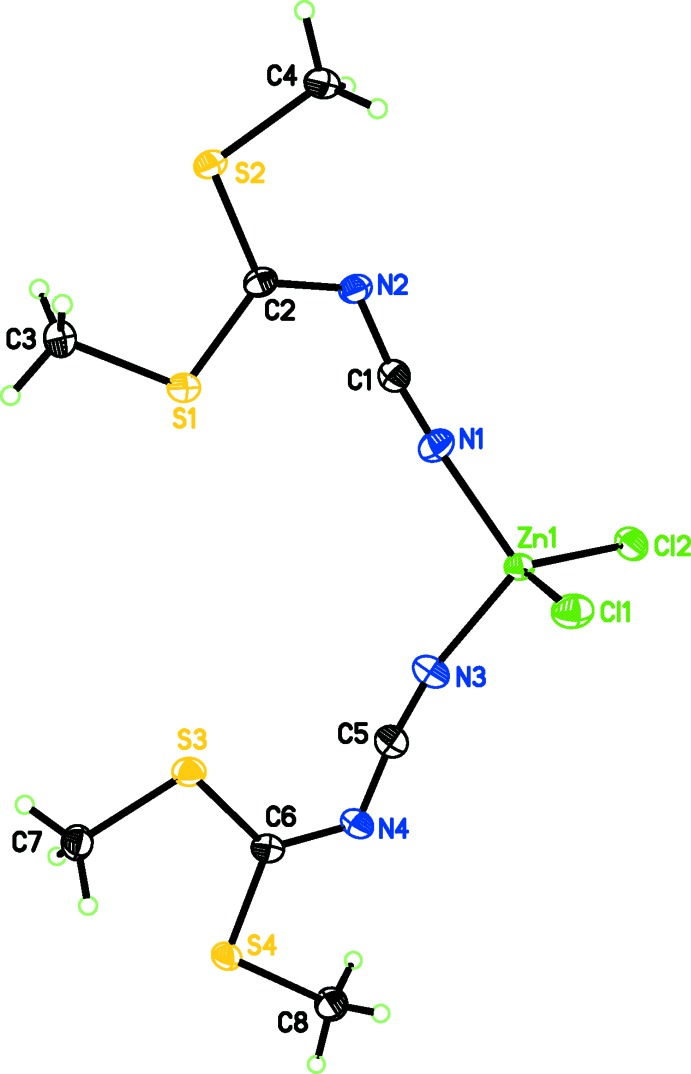
The mol­ecular structure of the title compound. Anisotropic displacement ellipsoids are depicted at the 50% probability level and H atoms as spheres of an arbitrary radius.

The structure of the title compound (Fig. 1 ▸) is isotypic with the Co complex reported recently (Diop *et al.*, 2016 ▸). The Zn^II^ atom is coordinated in a tetra­hedral fashion by two Cl atoms and the cyanide N atoms of two dimethyl *N*-cyano­dithio­imino­carbonate ligands. The Zn atom has a τ_4_ value of 0.94 (Yang *et al.*, 2007 ▸), indicating a near ideal tetra­hedral geometry (τ_4_ = 1 for ideal tetra­hedral and 0 for planar environments); τ_4_ = [360 - (α + β)]/141, where α and β are the two largest tetra­hedral angles.

## Supra­molecular features   

**Figure 2:**
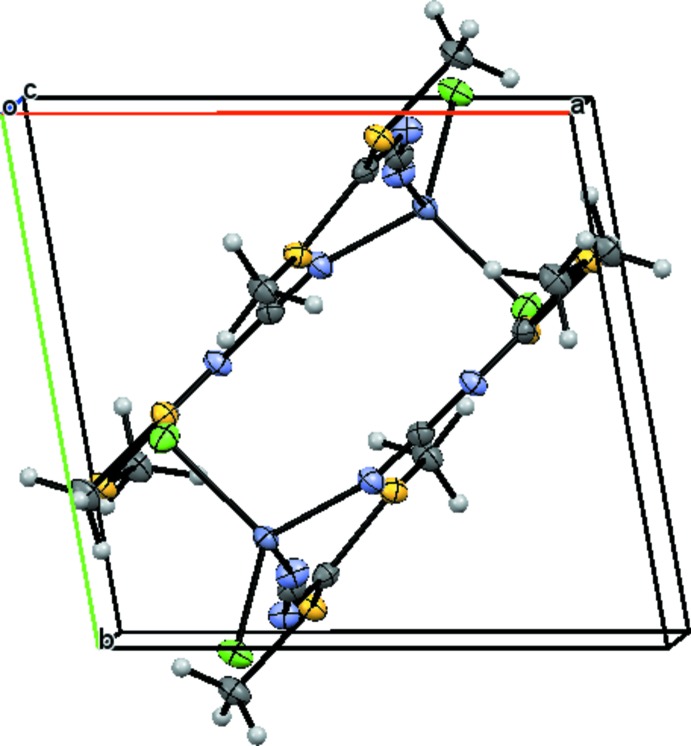
Packing diagram of the title compound, viewed approximately along the *c* axis, showing a pair of mol­ecules. Displacement ellipsoids are as in Fig. 1 ▸.

**Figure 3:**
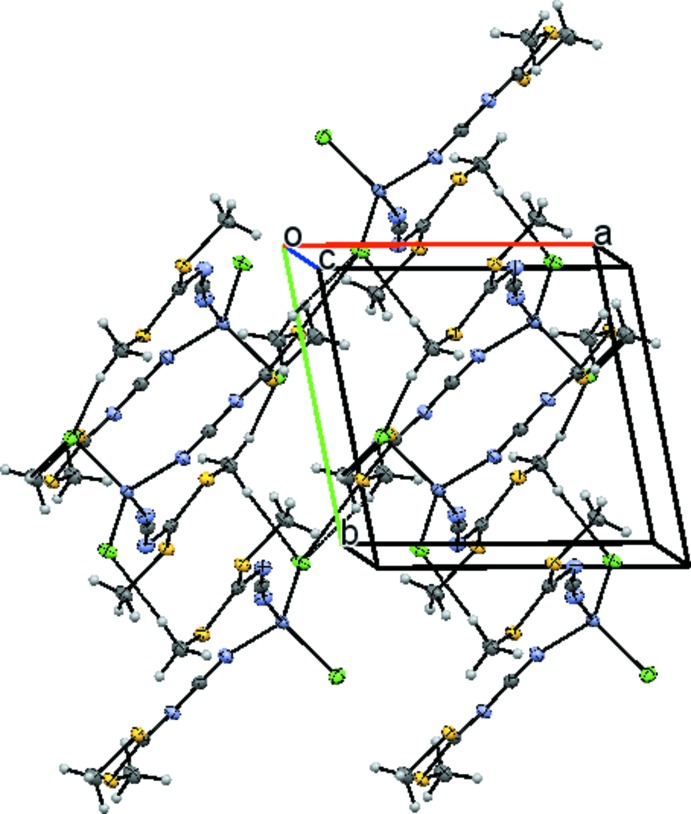
Packing diagram of the title compound viewed approximately along the *c* axis. Displacement ellipsoids are as in Fig. 1 ▸.

In the crystal, weak C—H⋯Cl hydrogen bonds (C3—H3*B*⋯Cl1^ii^ and C7—H7*B*⋯Cl1^ii^; Table 1 ▸) link the mol­ecules into inversion dimers (Fig. 2 ▸). The dimers are connected through a C4—H4*B*⋯Cl2^i^ hydrogen bond (Table 1 ▸) and an S2⋯Cl2^i^ short contact [3.3812 (7) Å], giving infinite chains along [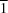
10]. These chains are then connected through a longer hydrogen bond (C7—H7*A*⋯Cl2^ii^) and an S4⋯Cl2^iv^ contact [3.3765 (7) Å; symmetry code: (iv) *x*, *y*, *z* + 1], leading to a layer parallel to the *ab* plane (Fig. 3 ▸).

## Synthesis and crystallization   

All chemicals are purchased from Aldrich Company, Germany and used as received. Dimethyl cyano­carbonimidodi­thio­ate was mixed in aceto­nitrile with ZnCl_2_ in a 1:1 ratio. Colourless block-like single crystals suitable for X-ray diffraction were obtained after a slow solvent evaporation at room temperature (303 K).

## Refinement   

Crystal data, data collection and structure refinement details are summarized in Table 2 ▸. The structure was solved by incorporating the coordinates from the isotypic compound [Co((MeS)_2_CNCN)_2_Cl_2_] (Diop *et al.*, 2016 ▸). Methyl H atoms were modeled as riding, with C—H = 0.98 Å and with *U*_iso_(H) = 1.5*U*_eq_(C), and were allowed to rotate to minimize their contribution to the electron density.

## Supplementary Material

Crystal structure: contains datablock(s) I. DOI: 10.1107/S2056989016002607/is5443sup1.cif

Structure factors: contains datablock(s) I. DOI: 10.1107/S2056989016002607/is5443Isup2.hkl

CCDC reference: 1453191

Additional supporting information:  crystallographic information; 3D view; checkCIF report

## Figures and Tables

**Table 1:** Hydrogen-bond geometry (Å, °)

*D*—H⋯*A*	*D*—H	H⋯*A*	*D*⋯*A*	*D*—H⋯*A*
C4—H4*B*⋯Cl2^i^	0.98	2.73	3.4486 (18)	131
C3—H3*B*⋯Cl1^ii^	0.98	2.80	3.5868 (19)	137
C7—H7*A*⋯Cl2^iii^	0.98	2.74	3.7165 (18)	176
C7—H7*B*⋯Cl1^ii^	0.98	2.84	3.5976 (18)	134

Symmetry codes: (i) 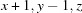; (ii) 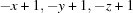; (iii) 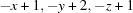.

**Table 2:** Experimental details

Crystal data	
Chemical formula	[Zn(C_4_H_6_N_2_S_2_)_2_Cl_2_]
*M*_r_	428.73
Crystal system, space group	Triclinic, *P* 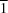
Temperature (K)	120
*a*, *b*, *c* (Å)	8.8574 (5), 8.8833 (6), 11.2391 (7)
α, β, γ (°)	73.0839 (16), 87.4301 (16), 79.9801 (16)
*V* (Å^3^)	833.14 (9)
*Z*	2
Radiation type	Mo *K*α
μ (mm^−1^)	2.29
Crystal size (mm)	0.49 × 0.21 × 0.17

Data collection	
Diffractometer	Bruker Kappa X8 APEXII
Absorption correction	Numerical (*SADABS*; Krause *et al.*, 2015 ▸)
*T*_min_, *T*_max_	0.520, 0.793
No. of measured, independent and observed [*I* > 2σ(*I*)] reflections	13605, 4239, 3939
*R*_int_	0.019
(sin θ/λ)_max_ (Å^−1^)	0.673

Refinement	
*R*[*F* ^2^ > 2σ(*F* ^2^)], *wR*(*F* ^2^), *S*	0.025, 0.062, 1.16
No. of reflections	4239
No. of parameters	176
H-atom treatment	H-atom parameters constrained
Δρ_max_, Δρ_min_ (e Å^−3^)	0.71, −0.37

Computer programs: *APEX2* and *SAINT* (Bruker, 2015 ▸), *SHELXT2014* (Sheldrick, 2015*a* ▸), *SHELXL2014* (Sheldrick, 2015*b* ▸), *XP* in *SHELXTL* (Sheldrick, 2008 ▸) and *Mercury* (Macrae *et al.*, 2006 ▸).

## supplementary crystallographic information

### Crystal data

**Table:**

[Zn(C_4_H_6_N_2_S_2_)_2_Cl_2_]	*Z* = 2
*M**_r_* = 428.73	*F*(000) = 432
Triclinic, *P*1	*D*_x_ = 1.709 Mg m^−^^3^
*a* = 8.8574 (5) Å	Mo *K*α radiation, λ = 0.71073 Å
*b* = 8.8833 (6) Å	Cell parameters from 8988 reflections
*c* = 11.2391 (7) Å	θ = 2.3–28.6°
α = 73.0839 (16)°	µ = 2.29 mm^−^^1^
β = 87.4301 (16)°	*T* = 120 K
γ = 79.9801 (16)°	Block, colourless
*V* = 833.14 (9) Å^3^	0.49 × 0.21 × 0.17 mm

### Data collection

**Table:**

Bruker Kappa X8 APEXII diffractometer	4239 independent reflections
Radiation source: fine-focus sealed tube	3939 reflections with *I* > 2σ(*I*)
Graphite monochromator	*R*_int_ = 0.019
Detector resolution: 8.33 pixels mm^-1^	θ_max_ = 28.6°, θ_min_ = 1.9°
combination of ω and φ–scans	*h* = −11→11
Absorption correction: numerical (*SADABS*; Krause *et al.*, 2015)	*k* = −9→11
*T*_min_ = 0.520, *T*_max_ = 0.793	*l* = −14→15
13605 measured reflections	

### Refinement

**Table:**

Refinement on *F*^2^	Primary atom site location: isomorphous structure methods
Least-squares matrix: full	Secondary atom site location: difference Fourier map
*R*[*F*^2^ > 2σ(*F*^2^)] = 0.025	Hydrogen site location: inferred from neighbouring sites
*wR*(*F*^2^) = 0.062	H-atom parameters constrained
*S* = 1.16	*w* = 1/[σ^2^(*F*_o_^2^) + (0.0275*P*)^2^ + 0.4996*P*] where *P* = (*F*_o_^2^ + 2*F*_c_^2^)/3
4239 reflections	(Δ/σ)_max_ = 0.001
176 parameters	Δρ_max_ = 0.71 e Å^−^^3^
0 restraints	Δρ_min_ = −0.37 e Å^−^^3^

### Special details

**Table:**

Geometry. All e.s.d.'s (except the e.s.d. in the dihedral angle between two l.s. planes) are estimated using the full covariance matrix. The cell e.s.d.'s are taken into account individually in the estimation of e.s.d.'s in distances, angles and torsion angles; correlations between e.s.d.'s in cell parameters are only used when they are defined by crystal symmetry. An approximate (isotropic) treatment of cell e.s.d.'s is used for estimating e.s.d.'s involving l.s. planes.

### Fractional atomic coordinates and isotropic or equivalent isotropic displacement parameters (Å^2^)

**Table:**

	*x*	*y*	*z*	*U*_iso_*/*U*_eq_	
Zn1	0.31840 (2)	0.80108 (2)	0.23625 (2)	0.01602 (6)	
Cl1	0.16956 (5)	0.61543 (5)	0.29276 (4)	0.02386 (9)	
Cl2	0.23470 (5)	1.01274 (5)	0.08093 (4)	0.02331 (9)	
S1	0.83793 (5)	0.41654 (5)	0.37573 (4)	0.01844 (9)	
S2	0.97579 (4)	0.27942 (5)	0.17752 (4)	0.01855 (9)	
S3	0.53848 (5)	0.72562 (5)	0.68555 (4)	0.01877 (9)	
S4	0.35318 (5)	0.94788 (5)	0.80760 (4)	0.01798 (9)	
N1	0.52396 (16)	0.69240 (17)	0.19696 (14)	0.0216 (3)	
N2	0.73637 (16)	0.50808 (17)	0.14285 (13)	0.0186 (3)	
N3	0.34751 (18)	0.87159 (18)	0.38602 (13)	0.0223 (3)	
N4	0.31171 (17)	0.95551 (17)	0.57596 (13)	0.0192 (3)	
C1	0.62644 (18)	0.60463 (19)	0.17805 (15)	0.0178 (3)	
C2	0.83921 (17)	0.41281 (18)	0.22362 (15)	0.0163 (3)	
C3	1.0004 (2)	0.2705 (2)	0.44393 (16)	0.0248 (3)	
H3A	0.9886	0.1666	0.4351	0.037*	
H3B	1.0075	0.2628	0.5323	0.037*	
H3C	1.0940	0.3023	0.4016	0.037*	
C4	0.9182 (2)	0.3114 (2)	0.01991 (15)	0.0230 (3)	
H4A	0.8123	0.2934	0.0184	0.034*	
H4B	0.9859	0.2369	−0.0164	0.034*	
H4C	0.9249	0.4213	−0.0285	0.034*	
C5	0.33745 (19)	0.90393 (19)	0.47793 (15)	0.0189 (3)	
C6	0.39261 (18)	0.88406 (19)	0.67851 (15)	0.0170 (3)	
C7	0.6027 (2)	0.6667 (2)	0.84453 (16)	0.0228 (3)	
H7A	0.6417	0.7550	0.8617	0.034*	
H7B	0.6845	0.5736	0.8586	0.034*	
H7C	0.5167	0.6393	0.9000	0.034*	
C8	0.1918 (2)	1.1031 (2)	0.75497 (17)	0.0237 (3)	
H8A	0.2209	1.1840	0.6817	0.036*	
H8B	0.1590	1.1526	0.8213	0.036*	
H8C	0.1075	1.0578	0.7330	0.036*	

### Atomic displacement parameters (Å^2^)

**Table:**

	*U*^11^	*U*^22^	*U*^33^	*U*^12^	*U*^13^	*U*^23^
Zn1	0.01630 (10)	0.01660 (10)	0.01569 (10)	0.00008 (7)	0.00063 (7)	−0.00723 (7)
Cl1	0.02395 (19)	0.0244 (2)	0.0270 (2)	−0.00800 (15)	0.00604 (15)	−0.01198 (16)
Cl2	0.0288 (2)	0.01982 (19)	0.01917 (18)	0.00453 (15)	−0.00438 (15)	−0.00668 (15)
S1	0.01807 (18)	0.02036 (19)	0.01693 (18)	−0.00120 (14)	0.00170 (14)	−0.00684 (14)
S2	0.01730 (18)	0.01884 (19)	0.01817 (18)	0.00245 (14)	0.00138 (14)	−0.00662 (14)
S3	0.02048 (18)	0.01800 (19)	0.01854 (19)	−0.00125 (14)	0.00216 (14)	−0.00781 (14)
S4	0.02055 (18)	0.01868 (19)	0.01489 (17)	0.00057 (14)	−0.00022 (14)	−0.00732 (14)
N1	0.0190 (7)	0.0186 (7)	0.0253 (7)	0.0010 (5)	0.0018 (5)	−0.0062 (6)
N2	0.0176 (6)	0.0185 (6)	0.0183 (6)	0.0011 (5)	0.0019 (5)	−0.0058 (5)
N3	0.0292 (7)	0.0225 (7)	0.0165 (7)	−0.0065 (6)	0.0013 (5)	−0.0069 (5)
N4	0.0243 (7)	0.0190 (7)	0.0150 (6)	−0.0034 (5)	0.0006 (5)	−0.0063 (5)
C1	0.0193 (7)	0.0165 (7)	0.0172 (7)	−0.0032 (6)	−0.0001 (6)	−0.0040 (6)
C2	0.0151 (7)	0.0153 (7)	0.0186 (7)	−0.0032 (5)	0.0031 (5)	−0.0052 (6)
C3	0.0242 (8)	0.0278 (9)	0.0189 (8)	0.0018 (7)	−0.0031 (6)	−0.0044 (7)
C4	0.0245 (8)	0.0267 (9)	0.0174 (8)	0.0016 (7)	0.0007 (6)	−0.0094 (6)
C5	0.0219 (8)	0.0165 (7)	0.0183 (7)	−0.0058 (6)	0.0011 (6)	−0.0036 (6)
C6	0.0193 (7)	0.0162 (7)	0.0168 (7)	−0.0052 (6)	0.0027 (6)	−0.0060 (6)
C7	0.0221 (8)	0.0236 (8)	0.0230 (8)	0.0017 (6)	−0.0043 (6)	−0.0097 (7)
C8	0.0256 (8)	0.0212 (8)	0.0229 (8)	0.0049 (6)	−0.0024 (6)	−0.0090 (7)

### Geometric parameters (Å, º)

**Table:**

Zn1—N3	2.0003 (15)	N3—C5	1.146 (2)
Zn1—N1	2.0016 (14)	N4—C5	1.308 (2)
Zn1—Cl2	2.2030 (4)	N4—C6	1.317 (2)
Zn1—Cl1	2.2210 (4)	C3—H3A	0.9800
S1—C2	1.7191 (17)	C3—H3B	0.9800
S1—C3	1.7922 (18)	C3—H3C	0.9800
S2—C2	1.7141 (16)	C4—H4A	0.9800
S2—C4	1.7935 (17)	C4—H4B	0.9800
S3—C6	1.7228 (17)	C4—H4C	0.9800
S3—C7	1.7980 (17)	C7—H7A	0.9800
S4—C6	1.7067 (16)	C7—H7B	0.9800
S4—C8	1.7914 (17)	C7—H7C	0.9800
N1—C1	1.144 (2)	C8—H8A	0.9800
N2—C1	1.309 (2)	C8—H8B	0.9800
N2—C2	1.315 (2)	C8—H8C	0.9800
			
N3—Zn1—N1	106.61 (6)	H3B—C3—H3C	109.5
N3—Zn1—Cl2	108.82 (5)	S2—C4—H4A	109.5
N1—Zn1—Cl2	110.62 (4)	S2—C4—H4B	109.5
N3—Zn1—Cl1	107.09 (4)	H4A—C4—H4B	109.5
N1—Zn1—Cl1	106.71 (4)	S2—C4—H4C	109.5
Cl2—Zn1—Cl1	116.505 (19)	H4A—C4—H4C	109.5
C2—S1—C3	103.78 (8)	H4B—C4—H4C	109.5
C2—S2—C4	101.50 (8)	N3—C5—N4	172.64 (19)
C6—S3—C7	103.40 (8)	N4—C6—S4	119.94 (13)
C6—S4—C8	101.30 (8)	N4—C6—S3	121.19 (12)
C1—N1—Zn1	166.17 (14)	S4—C6—S3	118.87 (9)
C1—N2—C2	120.32 (15)	S3—C7—H7A	109.5
C5—N3—Zn1	166.98 (14)	S3—C7—H7B	109.5
C5—N4—C6	120.40 (15)	H7A—C7—H7B	109.5
N1—C1—N2	172.98 (18)	S3—C7—H7C	109.5
N2—C2—S2	119.54 (12)	H7A—C7—H7C	109.5
N2—C2—S1	121.40 (12)	H7B—C7—H7C	109.5
S2—C2—S1	119.05 (9)	S4—C8—H8A	109.5
S1—C3—H3A	109.5	S4—C8—H8B	109.5
S1—C3—H3B	109.5	H8A—C8—H8B	109.5
H3A—C3—H3B	109.5	S4—C8—H8C	109.5
S1—C3—H3C	109.5	H8A—C8—H8C	109.5
H3A—C3—H3C	109.5	H8B—C8—H8C	109.5
			
C1—N2—C2—S2	176.35 (12)	C5—N4—C6—S4	−177.58 (12)
C1—N2—C2—S1	−2.5 (2)	C5—N4—C6—S3	2.2 (2)
C4—S2—C2—N2	−3.57 (15)	C8—S4—C6—N4	2.39 (15)
C4—S2—C2—S1	175.32 (10)	C8—S4—C6—S3	−177.42 (10)
C3—S1—C2—N2	−178.77 (14)	C7—S3—C6—N4	−176.93 (14)
C3—S1—C2—S2	2.36 (12)	C7—S3—C6—S4	2.89 (12)

### Hydrogen-bond geometry (Å, º)

**Table:**

*D*—H···*A*	*D*—H	H···*A*	*D*···*A*	*D*—H···*A*
C4—H4*B*···Cl2^i^	0.98	2.73	3.4486 (18)	131
C3—H3*B*···Cl1^ii^	0.98	2.80	3.5868 (19)	137
C7—H7*A*···Cl2^iii^	0.98	2.74	3.7165 (18)	176
C7—H7*B*···Cl1^ii^	0.98	2.84	3.5976 (18)	134

Symmetry codes: (i) *x*+1, *y*−1, *z*; (ii) −*x*+1, −*y*+1, −*z*+1; (iii) −*x*+1, −*y*+2, −*z*+1.

## References

[bb1] Bruker (2015). *APEX2* and *SAINT*. Bruker–Nonius AXS, Madison, Wisconsin, USA.

[bb2] Diop, M. B., Diop, L. & Oliver, A. G. (2016). *Acta Cryst.* E**72**, 66–68. 10.1107/S2056989015023439 PMC4704751 26870588

[bb3] Kojić-Prodić, B., Kiralj, R., Zlata, R. & Šunjić, V. (1992). *Vestn. Slov. Kem. Drus. (Bull. Slovenian Chem. Soc.)*, **39**, 367–381.

[bb4] Krause, L., Herbst-Irmer, R., Sheldrick, G. M. & Stalke, D. (2015). *J. Appl. Cryst.* **48**, 3–10. 10.1107/S1600576714022985 PMC4453166 26089746

[bb9] Macrae, C. F., Edgington, P. R., McCabe, P., Pidcock, E., Shields, G. P., Taylor, R., Towler, M. & van de Streek, J. (2006). *J. Appl. Cryst.* **39**, 453–457.

[bb5] Sheldrick, G. M. (2008). *Acta Cryst.* A**64**, 112–122. 10.1107/S0108767307043930 18156677

[bb6] Sheldrick, G. M. (2015*a*). *Acta Cryst.* A**71**, 3–8.

[bb7] Sheldrick, G. M. (2015*b*). *Acta Cryst.* C**71**, 3–8.

[bb8] Yang, L., Powell, D. R. & Houser, R. P. (2007). *Dalton Trans.* pp. 955–964. 10.1039/b617136b 17308676

